# Investigation of *CACNA1I* Cav3.3 Dysfunction in Hemiplegic Migraine

**DOI:** 10.3389/fnmol.2022.892820

**Published:** 2022-07-19

**Authors:** Neven Maksemous, Claire D. Blayney, Heidi G. Sutherland, Robert A. Smith, Rod A. Lea, Kim Ngan Tran, Omar Ibrahim, Jeffrey R. McArthur, Larisa M. Haupt, M. Zameel Cader, Rocio K. Finol-Urdaneta, David J. Adams, Lyn R. Griffiths

**Affiliations:** ^1^Genomics Research Centre, The Centre for Genomics and Personalised Health, School of Biomedical Sciences, Queensland University of Technology, Brisbane, QLD, Australia; ^2^Illawarra Health and Medical Research Institute, University of Wollongong, Wollongong, NSW, Australia; ^3^Weatherall Institute of Molecular Medicine, University of Oxford, Oxford, United Kingdom

**Keywords:** hemiplegic migraine, familial hemiplegic migraine, migraine genetics, ion channel, *CACNA1I*, Cav3.3, T-type calcium channels, voltage gated calcium channels

## Abstract

Familial hemiplegic migraine (FHM) is a severe neurogenetic disorder for which three causal genes, *CACNA1A*, *SCN1A*, and *ATP1A2*, have been implicated. However, more than 80% of referred diagnostic cases of hemiplegic migraine (HM) are negative for exonic mutations in these known FHM genes, suggesting the involvement of other genes. Using whole-exome sequencing data from 187 mutation-negative HM cases, we identified rare variants in the *CACNA1I* gene encoding the T-type calcium channel Cav3.3. Burden testing of *CACNA1I* variants showed a statistically significant increase in allelic burden in the HM case group compared to gnomAD (OR = 2.30, *P* = 0.00005) and the UK Biobank (OR = 2.32, *P* = 0.0004) databases. Dysfunction in T-type calcium channels, including Cav3.3, has been implicated in a range of neurological conditions, suggesting a potential role in HM. Using patch-clamp electrophysiology, we compared the biophysical properties of five Cav3.3 variants (p.R111G, p.M128L, p.D302G, p.R307H, and p.Q1158H) to wild-type (WT) channels expressed in HEK293T cells. We observed numerous functional alterations across the channels with Cav3.3-Q1158H showing the greatest differences compared to WT channels, including reduced current density, right-shifted voltage dependence of activation and inactivation, and slower current kinetics. Interestingly, we also found significant differences in the conductance properties exhibited by the Cav3.3-R307H and -Q1158H variants compared to WT channels under conditions of acidosis and alkalosis. In light of these data, we suggest that rare variants in *CACNA1I* may contribute to HM etiology.

## Introduction

Voltage-gated calcium (Cav) channels are widely expressed throughout the nervous system where their dysfunction can lead to a variety of neurological disorders including epilepsy, ataxia, and hemiplegic migraine (HM). HM is a rare severe subtype of migraine with aura coupled with reversible motor weakness, visual, sensory, and/or speech/language symptoms (Goadsby and Evers, 2020). The familial form, familial hemiplegic migraine (FHM), is usually inherited in an autosomal dominant manner, with pathogenic mutations in three main genes encoding cation transport proteins considered causal: *CACNA1A*, which encodes the pore-forming subunit of the Cav channel Cav2.1 (Ophoff et al., 1997); *SCN1A*, which encodes the pore-forming voltage-gated sodium channel Nav1.1; and *ATP1A2*, encoding the alpha-2 isoform of the Na^+^/K^+^-ATPase pump (Russell and Ducros, 2011).

Numerous causal mutations in the three FHM genes have been reported (de Vries et al., 2009; Friedrich et al., 2016). The full exonic analysis using next-generation sequencing (NGS) approaches has allowed more comprehensive studies of these genes. Nevertheless, while accounting for some HM cases, particularly familial cases with severe phenotypes, we and others have found that <20% of clinically diagnosed cases have pathogenic variants in the FHM genes (Hiekkala et al., 2018; Maksemous et al., 2019). Mutations in other genes including *PRRT2*, *PKND*, *ATP1A3*, *SLC1A3*, *SLC2A1*, and *SLC4A4* can cause disorders with overlapping symptoms and have been implicated in some cases that present with HM (Sutherland et al., 2019; Riant et al., 2022). Nevertheless, despite additional targeted analysis of whole-exome sequencing (WES) data for likely pathogenic variants in these genes, the majority of suspected HM cases remain genetically unsolved (Pelzer et al., 2018; Sutherland et al., 2020).

Familial hemiplegic migraine-causing mutations in *CACNA1A* are usually missense, and electrophysiological studies show gain-of-function (GOF) effects leading to channel hyperactivity (Hans et al., 1999; Tottene et al., 2002). The Cav channels control calcium influx in excitable membranes, initiating a wide range of calcium-dependent processes such as muscle contraction, release of neurotransmitters, gene expression to control neuronal excitability, and synaptic plasticity (Wadel et al., 2007; Simms and Zamponi, 2014). Amid the multitude of ion channels, mutations in different Cav channels may potentially be involved in the development of HM.

Cav channels fall into two major groups: high voltage-activated (HVA) comprising L-, N-, P/Q- (which includes *CACNA1A*), and R-types; and low-voltage-activated (LVA) T-type calcium channels. Compared with the HVA, LVA channels activate at more hyperpolarized potentials (or “lower” voltages), have faster inactivation, and have slower deactivation (Armstrong and Matteson, 1985; Bean, 1985). Thus, along with having very small single-channel conductance, their opening is regarded as “transient” (hence T-type). There are three T-type isoforms Cav3.1/α1G, Cav3.2/α1H, and Cav3.3/α1I encoded by the *CACNA1G*, *CACNA1H*, and *CACNA1I* genes, respectively. All Cav3 channels activate and inactivate within voltages near the resting membrane potential, but have unique gating, inactivation, and deactivation properties; pharmacological profiles; and specific cellular and subcellular expression (Klockner et al., 1999; Kozlov et al., 1999; Iftinca, 2011). In the brain, these unique electrophysiological properties allow T-type channels to finely regulate neuronal excitability. Thus, they have been implicated in neurological functions including sensory processing, sleep, and hormone and neurotransmitter release, as well as consciousness and cortical arousal *via* the thalamocortical system (Cheong and Shin, 2013).

In humans, T-type channel mutations can cause or increase the risk for several neurological and neurodevelopmental disorders including epilepsy, cerebellar ataxia and atrophy, autism spectrum disorders, and schizophrenia (Lory et al., 2020; El Ghaleb et al., 2021). With a role in regulating the neuroexcitability and high expression of *CACNA1I* in the thalamus and the cortex, regions important for migraine generation (May and Burstein, 2019; Barbanti et al., 2020), disruption of normal Cav3.3 function has the potential to also trigger HM.

In this study, we report numerous missense *CACNA1I* variants in a large cohort of HM probands and show altered function on selected Cav3.3 variants, suggesting that mutations in *CACNA1I* may play a role in the etiology of HM.

## Materials and Methods

### Patients

Blood samples or purified DNA from a large cohort of 187 patients from Australia and New Zealand, who were clinically diagnosed by neurologists as having HM, were sent to the Genomics Research Centre (GRC), Queensland University of Technology (QUT) for molecular genetic testing of the known FHM genes. All the 187 cases tested negative for pathogenic variants in the known FHM genes: *CACNA1A, ATP1A2*, and *SCN1A*. Following clinical reporting, these patients were enrolled in research to identify a potential genetic cause for their symptoms. Twenty-one extended family members of nine cases were also recruited for segregation analysis (14 = affected and 7 = unaffected). A total of 208 individuals (131 women and 77 men) with a mean age at diagnosis of 32.9 years were included in the study.

### Whole Exome Sequencing of the Hemiplegic Migraine Cohort

The DNA for 187 index cases previously referred to the GRC for molecular genetics testing was obtained either by in-house use of the QIAGEN QIAcube (Venlo, Netherlands) or extracted and purified by the referring hospitals. DNA was extracted from peripheral blood of 21 extended family members recruited for a segregation analysis. A WES was performed using the genomic DNA from the 208 participants in this study. WES libraries were prepared using the Ion AmpliSeq Exome RDY library preparation kit (Catalog number: A38264, Revision C.0, ThermoFisher Scientific, Scoresby, VIC Australia) according to the manufacturer’s protocol. The libraries were sequenced on the Ion Proton sequencer or a GeneStudio S5 Plus sequencer (ThermoFisher Scientific, Scoresby, VIC, Australia). Sequence reads were aligned to the human reference genome (hg19); single-nucleotide variants and indels were called using the Ion Torrent Suite software v5.10.1. The WES produced an average of 168× base coverage depth for the 10 rare variants identified in *CACNA1I* in this study. The bam format file generated by the Torrent Suite was uploaded and visualized for human examination using the Integrative Genomics Viewer (IGV v2.3) software^1^. The Ion Reporter software 5.10 (ThermoFisher Scientific) was used to perform automated variant annotation and filtering. To select candidate variants, iterative filtering was performed to focus on rare variants that alter protein-coding regions and canonical splice sites with a focus on the *CACNA1I* (OMIM# 608230, NM_001003406) gene. An additional analytical pass was also undertaken using VCF-DART, our in-house variant assessment tool to confirm that all relevant variants were identified (Benton et al., 2019). In addition, patients with the rare *CACNA1I* variants were also assessed for whether they carry variants in other migraine-associated genes (Sutherland et al., 2020).

### Functional Consequence of Hemiplegic Migraine-Associated Cav3.3 Variants

#### Cell Culture and Transient Transfection

HEK293T cells expressing the SV40 large T antigen (ATCC^®^ CRL-3216, Manassas, VA United States) were cultured in Dulbecco’s modified Eagle’s medium (DMEM, ThermoFisher Scientific, Scoresby, VIC, Australia), supplemented with 10% heat-inactivated fetal bovine serum (FBS, Bovigen, Keilor East, VIC, Australia), 1% penicillin and streptomycin (Pen/Strep), and 1% GlutaMAX™ supplement (Thermo Fisher Scientific). Cells were kept in a humidified incubator at 37°C/5% CO_2_ and were passaged at ∼80% confluence following standard protocols. Plasmid DNA encoding wild-type (WT) human Cav3.3, as well as five Cav3.3 HM-associated variants – p.R111G, p.M128L, p.D302G, p.R307H, and p.Q1158H – in the UniProt ID: Q9P0X4 isoform 4 were generated in pcDNA3.1-C-(k)DYK (constructs synthesized by GenScript, HK). Note that Q1158H corresponds to Q1193H in isoform 1. HEK293T cells were transiently co-transfected with plasmid cDNAs encoding either WT or mutant Cav3.3 and green fluorescent protein (GFP) to mark the transfected cells using the calcium phosphate method (Kumar et al., 2019). Whole-cell patch clamp recordings were conducted from GFP-positive cells 24–48 h post-transfection for all channels.

#### Electrophysiology

Whole-cell patch clamp recording of transfected HEK293T cells was used to characterize the biophysical and electrophysiological properties of the WT and five HM-associated Cav3.3 variant channels to provide insights into the impact of the identified mutations on membrane excitability and cell function. Recordings were obtained using a MultiClamp 700B amplifier controlled by the Clampex11/Digidata1440A acquisition system (Molecular Devices, San Jose, CA, United States) and undertaken at 23–25°C. These experiments were completed post-transfection depending on the most consistent expression of each channel isoform during the 24–48 h interval. Cells were perfused with an extracellular solution containing (in mM): 110 NaCl, 10 CaCl_2_, 1 MgCl_2_, 5 CsCl, 30 TEA-Cl, 10 D-glucose, 10 HEPES (pH 7.4 adjusted with NaOH), and ∼310 mOsmol.kg-1. On experiments examining the effects of pH modulation of Cav3.3 currents under alkaline conditions, the extracellular solution was adjusted to pH 8.0 with NaOH. To study the effects of acidic conditions on Cav3.3 function, the extracellular solution was buffered with 10 mM MES and adjusted to pH 6.5 using NaOH. Fire-polished borosilicate pipettes (1–3 MΩ) were filled with an intracellular solution composed of (in mM) 140 K-gluconate, 5 NaCl, 2 MgCl_2_, 5 EGTA, 10 HEPES (pH 7.2 adjusted with K-OH), and ∼285 mOsmol.kg–1.

The channel protein expression levels at the plasma membrane of all constructs were assessed by measuring peak current amplitudes (I_peak_ in pA) elicited by a 100-ms pulse to –10 mV from a holding potential (*V*_*h*_) of –90 mV and dividing this value by the cell capacitance (pF), rendering a value for current density in pA/pF. The cell capacitance is proportional to the cell surface area and thus provides a measure of cell size (Zimmermann et al., 2006).

The cells were held at –90 mV during stimulation protocols to examine various biophysical aspects of the channel, including I–V steady-state inactivation, and recovery from inactivation protocols. During these protocols, linear membrane capacitive currents were electronically subtracted (P/N = 4) and series resistance was compensated (60–80%). Based on these first pass protocols, we then modified protocols to look at channel activation, inactivation, or deactivation during different pH conditions. The data generated from each experiment were presented as SuperPlots (Lord et al., 2020).

#### Immunofluorescence

HEK293T cells were transfected with plasmids bearing C-terminally flag tagged Cav3.3 constructs and plated onto glass coverslips. Twenty-four hours post-transfection, the cells were fixed with 4% paraformaldehyde in PBS for 15 min, permeabilized (0.1% Triton X-100, 5 min), and blocked (PBS, 2% goat serum, and 2% BSA) for 30 min. Incubation with primary monoclonal ANTI-FLAG^®^ M2 antibody produced in mouse (1:1,000, F3165 Sigma-Aldrich) in blocking solution for 2 h at room temperature (RT) was followed by three washes (PBS) and stained with fluorophore-conjugated secondary antibody Alexa goat anti-mouse 488 (1:1,000, Alexa goat anti-mouse 488 Thermo Fisher) for 1 h at RT, further washed three times (PBS), and counterstained with DAPI. Coverslips were mounted on glass slides in Dako Mounting Medium (Agilent) and sealed. Images were acquired on an LSM 900 Airyscan 2 cryo confocal microscope (ZEISS) and visualized using Zen Lite (ZEISS) and ImageJ (Schindelin et al., 2012).

### Statistical Analysis

#### Whole-Exome Sequencing Statistical Analysis

Burden testing for the *CACNA1I* protein-coding variants in the HM cohort (MAF < 0.01) was performed by (a) summing the number of alternate alleles across variants per subject and then (b) calculating the total number of alleles in the cohort. These HM counts were then compared to count data derived from two general population control cohorts (i.e., gnomAD and UK Biobank). For the gnomAD population, we first removed any individuals with known neurological conditions to obtain a closer approximation to a control population. We also selected non-Finnish Europeans (NFE) to more closely match the ancestral background of the HM population in Australia. This resulted in ∼50,000 subjects for this comparison. Since individual-level data were not available, we estimated the allelic counts from the MAF for each variant. For the UK Biobank, we first removed all subjects that had self-reported headache symptoms and then focused only on the “British” cohort to better match the HM group. This resulted in ∼43,000 subjects. Individual-level count data were available for this cohort. The statistical significance of differences in allele counts (i.e., burden) was assessed using chi-square tests with a 1-tailed *p*-value. The justification for using 1-tailed *p*-value was that we are specifically hypothesizing an increased burden in HM cases compared to controls *a priori*. A *p*-value of –0.05 was used to assess statistical significance when comparing allele counts. These tests were performed using the R package (R-4.0.3).

#### Functional Study Statistical Analysis

Electrophysiological data are expressed as mean ± SEM and n is the number of cells. The Student’s unpaired *t*-tests were used to compare Cav3.3-WT with variant channels using GraphPad (GraphPad Software). Unpaired *t*-tests were used as each experiment was independent of one another. Statistical significance was set (*p* < 0.05) to compare WT and HM-associated Cav3.3 and enabled conclusions to be made surrounding hindered functionality of variant channels. Highly significant (*p* ≤ 0.001^***^), very significant (0.001 < *p* ≤ 0.01^**^), and significant (0.01 < *p* ≤ 0.05*) results were also differentiated. A minimum of *n* = 5 was used to calculate the statistical significance of various factors. Activation (1) and steady-state inactivation (2) curves were fit with the modified Boltzmann equations:


(1) $$G=1/\left(1+\exp\left(\frac{V\,m-V\,0.5}{d\,\left(x\right)}\right)\right)$$

(2) $$I=1-1/\left(1+\exp\left(\frac{V\,m-V\,0.5}{d\,\left(x\right)}\right)\right)$$

where *I* is the current, *G* is the conductance, *Vm* is the prepulse potential, *V*_0.5_ is the half-maximal activation or inactivation potential, and d(x) is the slope factor. Window currents (*I*_*W*_) were determined by calculating the area under the overlapping normalized mean activation and inactivation curves using OriginPro. Comparison of *I*_*W*_ of all channels assessed (*I*_*W*_Var) was obtained as the ratio against *I*_*W*_WT (*I*_*W*_Var/*I*_*W*_WT).

## Results

### Variants Identified in *CACNA1I* in the Hemiplegic Migraine Cohort Using Whole-Exome Sequencing

This study focused specifically on *CACNA1I* as a candidate for harboring functionally important mutations in HM. This is based on previous studies implicating *CACNA1I* mutations in neuronal excitability and resultant neurological disorders, coupled with high corticothalamic expression of *CACNA1I*, and potential connections to established migraine symptom-related neural pathways (Noseda et al., 2011). The genomic data for this gene were derived from a broader WES study of 187 HM cases negative for mutations in *CACNA1A*, *ATP1A2*, and *SCN1A* (Hiekkala et al., 2018; Maksemous et al., 2019). Briefly, Ion Reporter v5.10 was used to align reads to the hg19 genome assembly, call and annotate variants, which were also confirmed using our in-house analysis pipeline VCF-DART (Benton et al., 2019). *CACNA1I* is highly constrained with respect to missense variants (*Z* = 5.05) and extremely intolerant to loss of function (pLI = 1) (Lek et al., 2016). Variants in *CACNA1I* detected in the HM cohort were filtered for those with potential functional effects (altering amino acid composition of proteins, i.e., missense, predicted to affect splicing or result in stop-gain or loss); a total of 14 different exonic variants were detected, all of which were missense. An additional filtering to remove all variants found in the databases dbSNP^2^, 1000 Genomes Project^3^, and gnomAD^4^ at > 0.01 minor allele frequency (MAF) identified 10 rare variants. These were visually confirmed by the IGV software and validated by the Sanger sequencing. Thus, in total, 10 different heterozygous rare variants of interest in *CACNA1I* were confirmed in 17 individuals (Table 1), including four found in multiple unrelated probands: p.M128L was present in DGR1 and DGR239; p.A548T in DGR108 and DGR251; p.P991L in DGR94, DGR114, and DGR129; and p.Q1158H in DGR96, DGR161, DGR167, and DGR226. Clinical manifestations available for the cases (*n* = 9) with the five extracellular variants in *CACNA1I* are listed in Table 2.

**TABLE 1:** Rare functional variants (*n* = 10) identified in *CACNA1I* in 187 cases clinically diagnosed with HM.

Sample ID	Locus*	Amino acid change	Coding	SIFT	Polyphen2	LRT	Mutation taster	FATHMM	CADD	gnomAD exome_ NFE (MAF)	UKBiobank (MAF)	GRC HM- cases (MAF)	dbSNP
DGR211	chr22:39994250	p.R111G	c.331C > G	D	P	N	D	D	34	9.84E-06	2.23E-05	0.0026738	rs751729397
DGR1, DGR239	chr22:39996558	p.M128L	c.382A > C	T	B	D	N	D	9.094	0.0006	0.00071	0.00534759	rs58395851
DGR32	chr22:40037036	p.D302G	c.905A > G	T	B	U	N	D	23.6	0.0013	0.001	0.0026738	rs59635914
DGR246	chr22:40037051	p.R307H	c.920G > A	D	B	N	N	D	25.5	0.0073	0.0071	0.0026738	rs59986512
DGR108, DGR251	chr22:40045685	p.A548T	c.1642G > A	T	B	N	N	D	6.547	0.0019	0.00087	0.00534759	rs56859827
DGR206	chr22:40056424	p.G859C	c.2575G > T	T	P	U	N	D	24.6	9.04E-06	0	0.0026738	rs747381590
DGR72	chr22:40057233	p.P905L	c.2714C > T	T	B	N	D	D	24.2	1.81E-05	8.15E-05	0.0026738	rs376992678
DGR146	chr22:40057290	p.R924K	c.2771G > A	T	B	D	D	D	17.06	2.92E-05	2.33E-05	0.0026738	rs199552874
DGR94, DGR114, DGR129	chr22:40058145	p.P991L	c.2972C > T	T	B	U	N	D	8.374	0.007	0.0052	0.00802139	rs57299573
DGR96, DGR161, DGR167, DGR226	chr22:40059828	p.Q1158H	c.3474G > C	T	D	D	D	D	23.7	0.0041	0.0046	0.01069519	rs58500586

* GRCh37 (hg19) genome assembly.

*D, damaging or deleterious; P, possibly damaging; T, tolerated; B, benign; U, unknown; MAF, minor allele frequency; GRC, Genomics Research Centre. Transcript, CACNA1I (NM_001003406.2).*

**TABLE 2:** Clinical manifestation of nine cases with five extracellular variants in *CACNA1I*.

Amino acid change	Patients ID	Age	Gender	Clinical information
p.R111G	DGR211	5	M	Severe migraine and ataxia following head injury. Family history of migraine.
p.M128L	DGR1	54	F	Migraine, hemiplegia, vertigo, face sagging, loss of vision, confusion, clinical depression, relative had similar episode recently.
	DGR239	9	F	Two episodes of headache with right side weakness, self-resolved. Mother had similar episode recently
p.D302G	DGR32	58	F	Migraine, left side hemiplegia. Cognitive decline and progressive dementia, tremor when holding items, no parkinsonism, poor sleep, tiredness, angry and emotional
p.R307H	DGR246	34	F	Hemiplegic migraine
p.Q1158H	DGR161	9	F	Migraine coma, right sided hemiplegic migraine, past history of epilepsy since 3 years, mother has history of migraine, one episode of transient hemiplegia, maternal uncle said to have “headaches.”
	DGR167	16	M	Head injury induced migraine.
	DGR226	22	M	Hemiplegic Migraine.
	DGR96	40	F	Hemiplegic Migraine-stroke

*F, female; M, male.*

All confirmed variants were then subjected to *in silico* pathogenicity assessment using SIFT (Ng and Henikoff, 2001), PolyPhen2 (Adzhubei et al., 2010), LRT (Chun and Fay, 2009), MutationTaster (Schwarz et al., 2010), FATHMM (Shihab et al., 2013), and CADD (Kircher et al., 2014; Table 1). The *in silico* predictor assessment was somewhat variable; nevertheless, the variants p.R111G, p.R924K, and Q1158H all scored as deleterious in at least 3/6 of the predictor programs used.

Segregation analyses were performed for available family members by the Sanger sequencing. A segregation analysis of the p.M128L variant was performed in the proband (DGR1) and her daughter who suffer from severe migraine, confirming allele sharing between the two. The remainder of samples were unrelated probands with no other family samples available.

Notably, four of the probands that carry rare *CACNA1I* variants were also found to carry rare variants in other genes potentially associated with migraine: DGR211 (p.R111G, *CACNA1I*) had a variant annotated to be benign variant in *PRRT2* (p.P216L) and DGR32 (p.D302G, *CACNA1I*) carries a likely benign deletion variant in *PNKD* (p.M381_H382del). DGR161 and DGR96 (p.Q1158H, *CACNA1I*) were found to carry variants in *ATP1A4* (p.V146I and p.D685H), respectively (Sutherland et al., 2020), albeit the evidence for a role of this latter gene in migraine is still very limited.

A burden analysis for *CACNA1I* variants was performed by per-subject allele counting across the 10 rare variants (MAF < 0.01) and summing allele counts in both the case and control populations. The chi-square test with a one-tailed *p*-value was used to assess the statistical significance of the difference between groups (Table 1). The case group had a higher frequency of aggregated alternate variants compared to gnomAD, non-Finnish Europeans (gnomAD_NFE) controls (0.0045 vs. 0.0020), and UK Biobank controls (0.0045 vs. 0.0020). The increased burden was statistically significant using both control groups (*P* = 0.00005, OR = 2.3 and *P* = 0.0004, OR = 2.32, respectively).

### Functional Characterization of Hemiplegic Migraine-Associated Cav3.3 Variants

**FIGURE 1:**
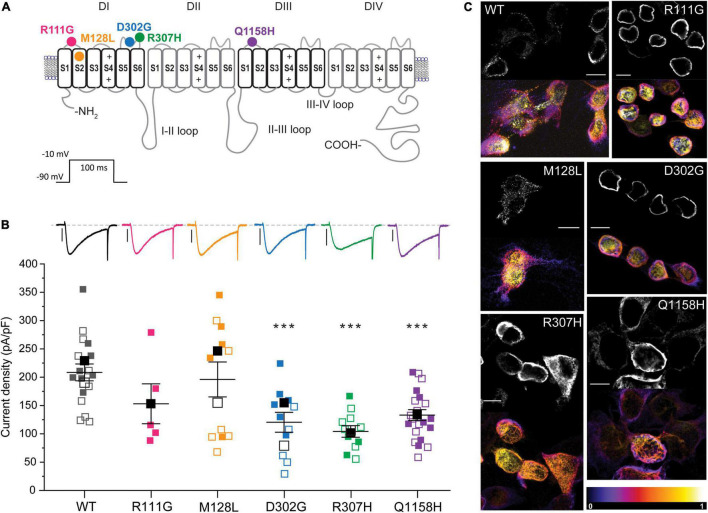
Location and functional expression of Cav3.3 HM-associated variants. **(A)** Schematic representation of the Cav3.3 channel protein and location of the HM-associated variants functionally characterized. Modular architecture of Cav3.3 showing the four homologous domains (DI–DIV). Each domain is composed of a voltage sensor module (transmembrane segments S1–S4) and a pore module (S5, a re-entrant pore loop and S6). The locations of the amino acids changed by HM-associated rare variants are marked with circles. Color scheme maintained hereafter: R111G (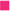), M128L (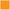), D302G (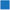), R307H (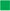), and Q1158H (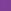). **(B)** Top: representative whole-cell currents from Cav3.3 WT and HM-associated variants R111G (fuchsia), M128L (orange), D302G (blue), R307H (green), and Q1158H (purple) expressed in HEK293T cells. Voltage-gated Ca^2+^ currents were elicited by a 100-ms pulse to −10 mV (*V*_*h*_: −90 mV, inset). Scale bars: 1 nA. Bottom: summary of current density calculated from peak current amplitude (I_peak_/cell capacitance in pA/pF). In this and subsequent SuperPlots (Lord et al., 2020), individual values and mean ± SEM (calculated from all data points in each group) are shown. Biological variabilities from independent transfections and recording time points are conveyed by different symbols. Within biological replicates, empty symbols correspond to determinations made 24 h post-transfection (□: mean 24 h), and solid symbols correspond to 48 h post-transfection recordings (■: mean 48 h). The statistical significance was determined through a paired Student’s *t*-test against Cav3.3 WT. *p* ≤ 0.001 (***), 0.001 < *p* ≤ 0.01 (**), or 0.01 < *p* ≤ 0.05 (*). **(C)** Confocal micrograph from immunostained Cav3.3 WT and variants (indicated by the corner labels) showing distinct expression patterns. Paired images of Alexa 488 fluorescence of center stack (top) and the depth-encoded full stack sets are shown for each construct. The color scale (bottom right) indicates relative distance from the coverslip. DAPI nuclear stain is not shown here for clarity, see Supplementary Figure 1A. Scale bar 10 microns.

To investigate whether variants identified in HM patients can elicit measurable changes in Cav3.3 channel function, we focused on the five variants that result in amino acid changes (p.R111G, p.M128L, p.D302G, p.R307H, and p.Q1158H) in structurally and/or functionally defined regions (i.e., extracellular or transmembrane domains) of Cav3.3 (Figure 1A). Four of the selected variants lay within Cav domain I (DI) and correspond to (1) a neutralizing change of the arginine (basic) to glycine (neutral) in position 111 (R111G, fuchsia) within the linker between transmembrane segments S1 and S2; (2) a conservative change of methionine to leucine in position 128 (M128L, orange) predicted to reside in the transmembrane segment S2 (eight amino acids downstream from the S1–S2 linker); (3) a neutralizing change from an aspartate (acidic) to a glycine in position 302 (D302G, blue); and (4) a semi-conservative substitution of arginine 307 to a histidine (R307H, green). The latter two changes occur within neighboring residues in DI’s S5–S6 Pore-Loop. The fifth HM variant selected for functional testing corresponds to a glutamine to histidine change in position 1158 (Q1158H, purple) within the DIII S1–S2 linker of the Cav3.3 protein.

The other *CAGNA1I* rare variants occurred in intracellular loops lacking distinctive structural features and were therefore not prioritized for functional testing in this study. Nevertheless, preliminary *in silico* analyses using MusiteDeep (Wang et al., 2020) identified potential disruptions to post-translational modifications (PTM) for variants p.A548T, p.G859C (loss of phosphorylation in adjacent serine residues), and p.R924K (appearance of a lysine acetylation site), verifying the presence of these novel PTMs would require extensive biochemical analyses beyond the scope of this study, and thus could form part of future work.

### Expression Level: Current Density and Immunostaining

The Cav3.3 channel sequence under UniProt ID: Q9P0X4 was considered the parental sequence (wild type, WT) in this study, from which the HM-associated variants identified were introduced by site-directed mutagenesis. Plasmids encoding WT and variants of Cav3.3 were transiently transfected into HEK293T cells and their functional expression was characterized by patch-clamp electrophysiology. The heterologous expression of all channel constructs in HEK293T cells yielded robust inward calcium currents (Figure 1B). To account for differences in expression between HEK293T cell passages and/or batches, the wild-type Cav3.3 channels were transfected and recorded in parallel with each of the assessed variants. This is portrayed in figures through the use of the same symbols for WT (black) and corresponding variant channels (according to color scheme) recorded from a given transfection day. The density of calcium currents mediated by Cav3.3 WT and HM variants was determined 24 h and/or 48 h post-transfection (Figure 1B; empty and filled symbols, respectively). In comparison to Cav3.3 WT (208.4 ± 14.9 pA/pF, *n* = 17), the current densities of variants D302G (120.5 ± 17.5 pA/pF, *n* = 11; *p* = 0.0008), R307H (104.2 ± 10.3 pA/pF, *n* = 11; *p* < 0.0001) and Q1158H (133.1 ± 9.6 pA/pF, *n* = 21; *p* < 0.0001) were significantly lower at both time points, while variants R111G (153.0 ± 35.2 pA/pF, *n* = 5; *p* = 0.1101) and M128L (196.0 ± 30.9 pA/pF, *n* = 11; *p* = 0.6902) were expressed at current levels comparable to WT (Figure 1B).

To assess the potential mechanisms underlying the current density profiles observed, C-terminally flag-tagged Cav3.3 WT and variant constructs transiently expressed in HEK293T cells were immunostained 24 h post-transfection using an anti-flag primary antibody (raised in mouse) and imaged in 3D by confocal microscopy. All constructs revealed positive immunostaining when probed with an Alexa 488 coupled anti-mouse secondary antibody (Figure 1C) attesting to the synthesis of the full-length protein products. The shown paired images include Alexa 488 fluorescence from the center stack (∼ cell equator, top) and depth-encoded projections of all the imaged stacks (bottom) per construct to enable the resolution of the z-plane (black corresponds to zero distance from the cover glass). Fake-colored orthogonal views of all presented images and primary antibody omitted control (WT) are included in Supplementary Figure 1. It can be observed that Cav3.3 WT transfected cells display distinctive punctate staining radiating away from the nucleus throughout the stack depth suggestive of predominant expression of the wild-type construct in the plasma membrane. In contrast, the staining of R111G and D302G variants appears as a continuous band in close proximity to the nuclear membrane (note strong white yellow signal near the cell nucleus, Figure 1C, and continuous green in orthogonal view from Supplementary Figure 1) consistent with the accumulation of these protein products within intracellular membranous organelles such as the endoplasmic reticulum. Notably, the current recordings for the Cav3.3-R111G variant were only possible 48 h post-transfection (see Supplementary Figure 1C for individual plots of data acquired at each time point), whereas abundant staining was evident at 24 h post-transfection suggesting potential impairments in recombinant protein trafficking for this variant in HEK293T cells. The expression pattern of M128L, H307R, and Q1158H revealed intermediate levels of punctate and band-like staining likely reflecting less-profound changes in protein trafficking for these variants.

### Voltage Dependence of Activation

**FIGURE 2:**
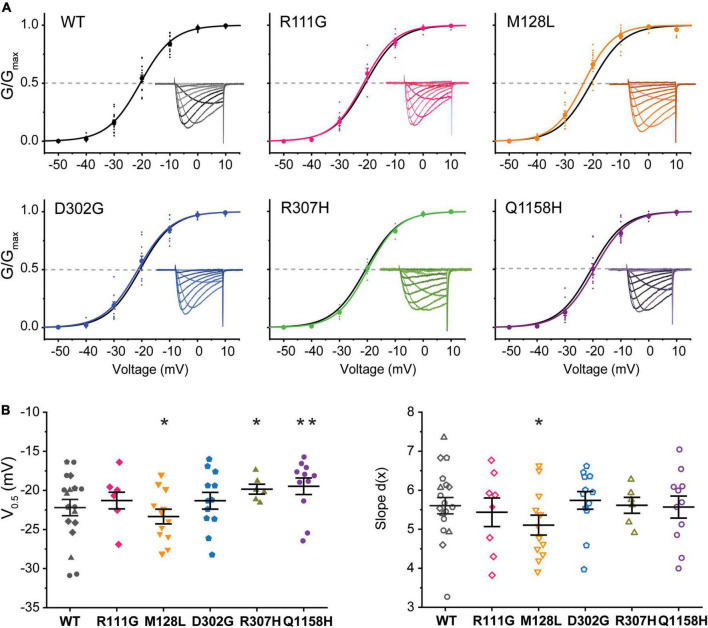
Voltage dependence of activation of Cav3.3 WT and HM-associated variants. **(A)** Normalized conductance (G/G_max_) vs. voltage relationships for Cav3.3 and HM variants. Representative current families are included as insets. Dotted lines are drawn at G/Gmax = 0.5 to highlight the voltage of half-maximal activation (activation V_0.5_). **(B)** SuperPlots of activation V_0.5_ and slope [d(x)]. Symbols represent determination made from cells transfected on the same day (i.e., biological replicates). 0.001 < *p* < 0.01 (**), 0.01 < *p* 0.05 (*).

The voltage dependence of activation of recombinant Cav3.3 WT and the five selected variants was evaluated from peak current amplitudes in response to 100-ms-long voltage steps from –90 to +60 mV (*V*_*h*_ = –90 mV) delivered at a frequency of 0.2 Hz. Representative current families for all channels are included as insets within the normalized conductance vs. voltage (G/Gmax-V) relationship plots generated for all Cav3.3 variants and WT as presented in Figure 2. The half-activation voltages (act V_0.5_) of Cav3.3-M128L (–23.3 ± 0.9 mV, *n* = 12; *p* = 0.0216) and Cav3.3-Q1158H (–17.6 ± .3 mV, *n* = 9; *p* = 0.0025) displayed small but statistically significant shifts from Cav3.3-WT activation (–20.6 ± 0.7 mV, *n* = 15). A very small depolarizing shift in the activation V_0.5_ of variant R307H (–19.8 ± 0.6 mV, *n* = 6; *p* = 0.04914) reached significance while no alterations were detected for R111G (–21.3 ± 1.1 mV, *n* = 8; *p* = 0.5709) and D302G (–21.3 ± 1.1 mV, *n* = 12; *p* = 0.5648) for this parameter.

Furthermore, currents mediated by all HM-associated Cav3.3 channel variants presented similar voltage dependence (slope) to WT-Cav3.3, with only M128L displaying minor differences in average slope (5.1 ± 0.3, *n* = 12) compared to WT channels (5.8 ± .2, *n* = 15; *p* = 0.0428). Thus, we find a small but significant alteration of the voltage dependence of activation for the M128L variant channel with a hyperpolarizing shift in act V_0.5_ and steeper current–voltage (I–V) slope compared to WT, as well as a significant depolarizing shift in act V_0.5_ for Q1158, without discernible changes to its voltage dependence (Figure 2B).

### Voltage Dependence of Steady-State Inactivation

**FIGURE 3:**
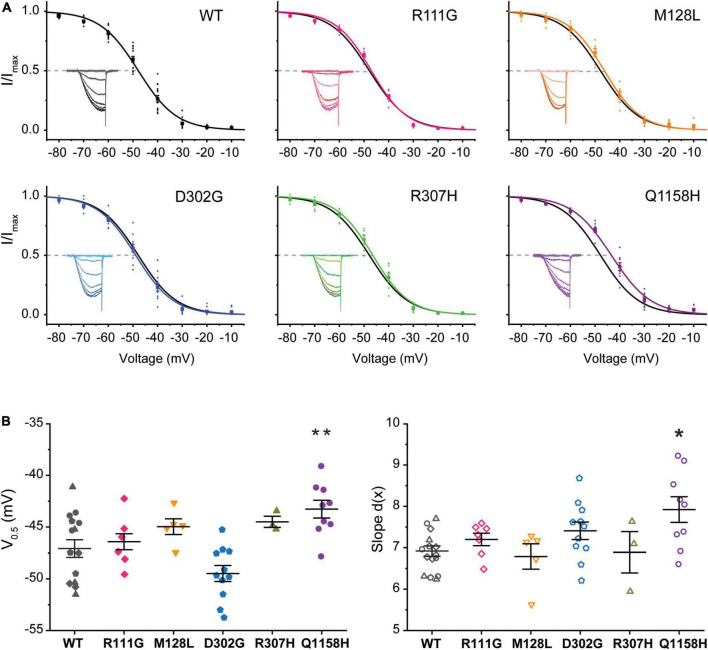
Steady-state inactivation (SSI) of HM-associated Cav3.3 variants. **(A)** Availability plots were constructed from normalized peak currents (I/I_*max*_) vs. the inactivating pre-pulse potential. Representative SSI current traces for each channel variant are shown in the insets. Gray dotted lines indicate the potential at which 50% of the channels are inactivated (inact V_0.5_). **(B)** SuperPlots summarizing SSI V_0.5_ and slope [d(x)] for all variants. 0.001 < *p* < 0.01 (**), 0.01 < *p* 0.05 (*).

A standard steady-state inactivation (SSI) stimulation protocol was implemented to determine the voltage dependence of SSI for each of the five Cav3.3 variant channels under study and compare it to WT. Representative Ca^2+^ current families used to build the availability plots for all the identified variants are presented as insets in Figure 3. The SSI stimulation protocol consisted of a 1-s-long pre-pulses from –90 to +20 mV to inactivate the Cav3.3 channels followed by a 50 ms square pulse to –20 mV (*V*_*h*_ = –90 mV) at.1 Hz to track the remaining channel population able to activate after the pre-pulses. Availability plots were fit using a Boltzmann equation (see section “Materials and Methods”) to extract the voltage-dependent parameters of SSI for WT and variant Cav3.3 channels. As shown in Figure 3, there were no significant differences in the voltage dependence of half maximal inactivation (inact V_0.5_) between WT Cav3.3 [V_0.5_ –47.1 ± .9 mV, d(x) 6.9 ± 0.1, *n* = 15] and R111G (–47.0 ± 0.5 mV, *n* = 7; *p* = 0.9639), M128L (–45.0 ± 0.6 mV, *n* = 5; *p* = 0.1873), D302G (–49.5 ± 0.8 mV, *n* = 11; *p* = 0.054), and R307H (–45.7 ± 1.1 mV, *n* = 6; *p* = 0.2057) variant channels. Concurrently, no changes to SSI’s slope component for the same four variant channels, R111G (7.2 ± 0.1, *n* = 7; *p* = 0.7035), M128L (6.8 ± 0.3, *n* = 5; *p* = 0.4554), D302G (7.4 ± 0.2, *n* = 11; *p* = 0.2662), and R307H (7.2 ± 0.3, *n* = 6; *p* = 0.7019), were observed in comparison to Cav3.3-WT inactivation slope (6.9 ± 0.1, *n* = 15).

Importantly, Cav3.3-Q1158H displayed a 3.8 mV depolarizing shift in SSI V_0.5_ (–43.3 ± 0.9 mV, *n* = 9; *p* = 0.0072) and shallower slope [*d*(x) 7.9 ± 0.3; *p* = 0.026] compared to WT Cav3.3 channels (Figure 3). Hence, under the same recording conditions, both activation and inactivation properties of the Cav3.3-Q1158H variant differ from those of the “reference” WT channel.

**FIGURE 4:**
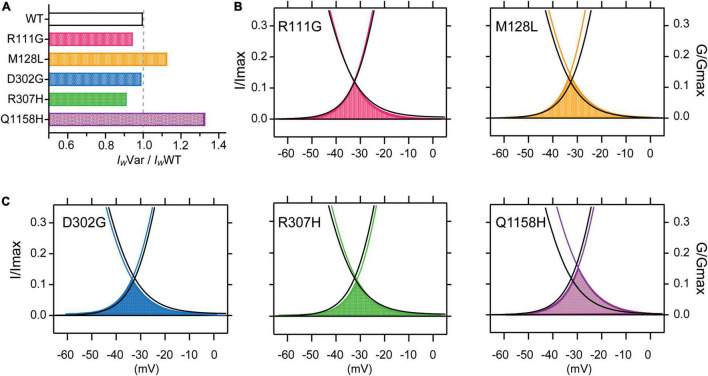
Cav3.3-WT and HM-associated variant window currents. The window current (*I*_*W*_) was determined from the area under the overlapping normalized activation and inactivation curves (AUC) of WT and HM-associated Cav3.3 variants (average traces from Figures 2, 3). **(A)** Total AUCs were divided by Cav3.3-WT AUC (*I*_*W*_Var/*I*_*W*_WT) for comparison. In **(B,C)** window currents are highlighted by the shading color corresponding to each HM-associated variant and plotted along Cav3.3 WT activation and SSI curves (in black).

It has been shown that a subpopulation of T-type channels remains tonically active within the membrane potential overlap between activation and inactivation voltages in what is called the window current (*I*_*w*_). The *I*_*w*_ is thus carried by a fraction of T-type channels that do not fully inactivate, allowing a steady influx of Ca^2+^ and concomitant tonic depolarization; Cav3.3 channels are the most distinctive of the T-type family displaying the slowest activation and inactivation kinetics and larger window currents (Klockner et al., 1999; Crunelli et al., 2005). Window currents (*I*_*W*_) were determined from the area under the overlapping normalized mean activation and inactivation curves obtained in this study (Figures 2, 3). A comparison of *I*_*W*_ of all channels assessed (*I*_*W*_Var) was obtained as the ratio against *I*_*W*_WT (*I*_*W*_Var/*I*_*W*_WT). The window currents of all variants analyzed did not overlap with those of Cav3.3 WT channels. Even though, D302G channels displayed window currents with an area that matched those of WT channels (*I*_*W*_D302G/*I*_*W*_WT = .99, Figure 4A), a small leftwards shift was apparent (Figure 4C, blue). Both R111G and D307H revealed a somewhat decreased availability as extrapolated from relative window current ratios of 0.95 and 0.91, respectively (Figures 4A–C), whereas Cav3.3-M128L (*I*_*W*_M128L/*I*_*W*_WT = 1.13) and -Q1158H (*I*_*W*_Q1158H/*I*_*W*_WT = 1.33) window currents were larger 10–30% larger (Figure 4A, yellow and purple, respectively). Furthermore, and consistent with the hitherto reported voltage-dependent parameters, Q1158H channels also evidenced a depolarizing shift in the voltage range of its window current compared to WT Cav3.3 channels (Figure 4C, purple).

### Current Kinetics

**FIGURE 5:**
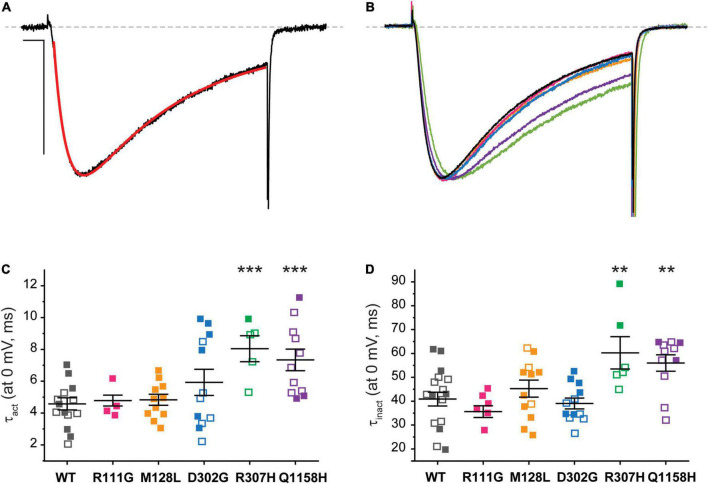
Cav3.3-WT and HM-associated variant current kinetics. **(A)** The speed of Ca^2+^ current activation (τ_*act*_) and inactivation (τ_inact_) during 100-ms stimuli (inset) was evaluated by fitting (red) the elicited currents with an exponential product equation (bottom), where **(C)** is the *y*-intercept. Scale bars: 10 ms, 500 pA. **(B)** Representative currents from Cav3.3 variants studied. **(C)** Summary of time constants of current activation (τ_*act*_). **(D)** Summary of time constants of current inactivation (τ_inact_). *p* < 0.001 (***), 0.001 < *p* < 0.01 (**).

The current kinetics of each channel variant were compared to those of Cav3.3 WT and shown in Figure 5. The steady state currents elicited by a 100-ms depolarization to 0 mV (*V*_*h*_ −90 mV, 0.1 Hz) were averaged and approximated through exponential fits to derive time constants for activation (τ_*act*_) and inactivation (τ_inact_), as shown for Cav3.3 WT in Figure 5A (see section “Materials and Methods”). For ease of comparison, representative current traces from all Cav3.3 variants were normalized to their peak amplitude and superimposed (Figure 5B). There were no discernible differences when comparing the activation time constant between Cav3.3-WT (τ_*act*_ = 4.6 ± 0.4 ms, *n* = 18) and variants R111G (4.8 ± 0.3 ms, *n* = 6; *p* = 0.7596), M128L (τ_*act*_ = 4.8 ± 0.4 ms, *n* = 11; *p* = 0.06453), and D302G (5.9 ± 0.8 ms, *n* = 12; *p* = 0.1228) (Figure 5C). Similarly, there were no apparent deviations from WT τ_inact_ (40.9 ± 2.9 ms, *n* = 18) with respect to R111G (35.6 ± 2.4 ms, *n* = 8; *p* = 0.2713), M128L (45.3 ± 3.6 ms, *n* = 12; *p* = 0.3471), and D302G (39.0 ± 2.2, *n* = 12; *p* = 0.6472) (Figure 5D).

In contrast, currents mediated by the Cav3.3-R307H (green) and -Q1158H (purple) channels displayed slower open channel kinetics than those mediated by WT channels (Figures 5B–D). Thus, the activation time constants of R307H (τ_*act*_ = 8.0 ± 0.7 ms, *n* = 6; *p* = 0.0002) and Q1158H (τ_*act*_ = 7.3 ± .7 ms, n = 11; *p* = .0009) were ∼2-fold slower than WT’s τ_*act*_. Whereas, R307H (τ_inact_ = 60.3 ± 6.8 ms, *n* = 6; *p* = 0.0055) and Q1158H (τ_inact_ = 56.0 ± 3.4 ms, *n* = 11; *p* = 0.0027) both displayed ∼1.5-fold slower inactivation kinetics in comparison to those of WT (Figures 5C,D).

### Extracellular pH Modulation of Cav3.3-WT and Variants R307H and Q1158H

**FIGURE 6:**
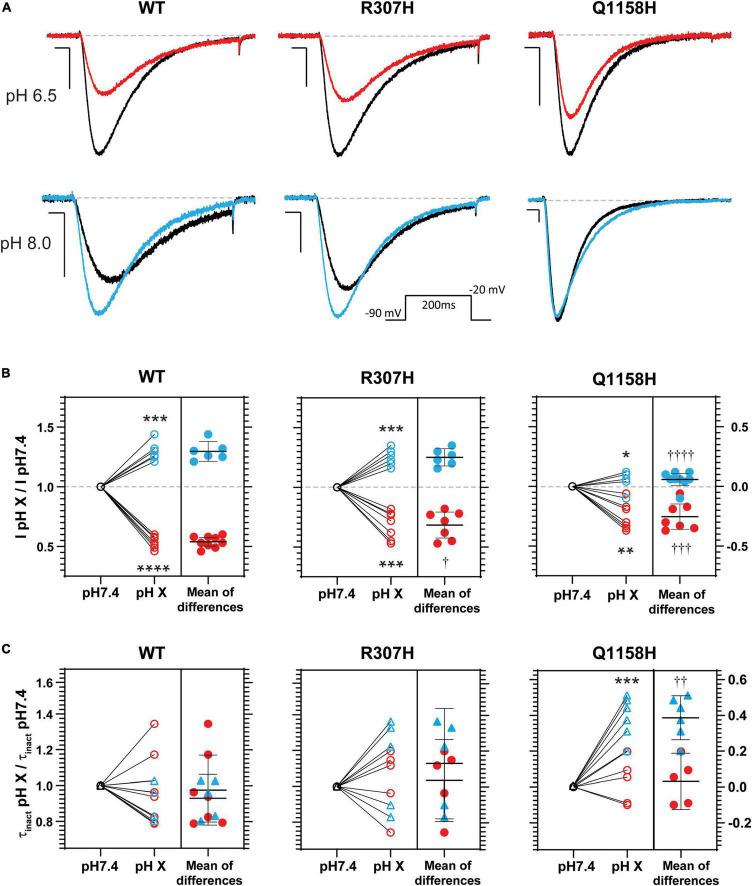
pH_*o*_ modulation of Cav3.3- WT-, - R307H-, and -Q1158H-mediated currents. **(A)** Representative Ca^2+^ current traces in response to 200-ms depolarizing pulses to −20 mV (*V*_*h*_ = −90 mV, 0.1 Hz, inset) recorded in control (black, pH 7.4), acidic (red, pH 6.5), or alkaline (blue, pH 8.0) pH_*o*_ conditions. Scale bars: 10 ms, 200 pA. Dashed line indicates zero level. **(B)** Relative change in peak current amplitude (I pH X/I pH7.4) for WT and HM-associated Cav3.3 variants (pH 7.4: ○; pH 6.5: 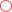; pH 8.0: 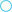; difference between pH 6.5: 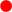, or pH 8.0: 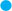). **(C)** Relative changes in inactivation time constant (τ_inact_ pH X/τ_inact_ pH7.4) for WT and HM-associated Cav3.3 variants (pH 7.4: ○;, △; pH 6.5: 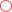; pH 8.0: 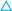; difference between pH 6.5: 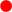, or pH 8.0: 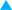). In **(B,C)** paired *t*-test for pH 7.4 vs. pH X: *p ≤ 0.05; **p ≤ 0.05; ***p < 0.005; ****p < 0.0005. One-way ANOVA with Tukey’s multiple comparisons test for WT vs. variant: ^†^*p* ≤ 0.05; ^††^*p* ≤ 0.05; ^†††^*p* ≤ 0.005; ^††††^*p* ≤ 0.0001.

Histidine residues bear a partial charge at physiological pH values (isoelectric point pH 7.6) and therefore changes in pH affect its protonation/charge. Thus, histidine residues act as [H^+^] sensors that, when present in exposed functional domains, have modulatory effects on voltage-gated ion channel function (Claydon et al., 2000; Finol-Urdaneta et al., 2006; Jones et al., 2013; Huang et al., 2020). Since variants R307H and Q1158H involve histidine substitutions within extracellular domains, the effects of changes in extracellular pH (pH_*o*_) on Cav3.3 WT-mediated currents (amplitude, τ_inact_ and act V_0.5_) were investigated and compared to the hereby identified histidine-bearing novel HM-associated variants. Cav3.3 currents elicited by repeated pulse stimulation (200 ms, −20 mV, *V*_*h*_ = −90 mV, 0.1 Hz) were recorded at neutral pH_*o*_ (pH 7.4) and during transition to defined acidic (pH 6.5) or alkaline (pH 8.0) conditions (Figure 6). WT Cav3.3 currents were strongly inhibited at acidic pH_*o*_ with an average relative peak current inhibition of 46% (fractional current remaining at pH 6.5, I_*pH6.5*_/I_*pH7.4*_ = 0.54 ± 0.02; *n* = 9; paired *t*-test *p* < 0.0001) while the alkalinization (pH 8.0) of the extracellular media lead to a 30% increase in peak current (I_*pH8.05*_/I_*pH7.4*_ = 1.29 ± 0.033; *n* = 6, paired *t*-test *p* = 0.0003) (Figure 6B, left). In contrast, exposure to acidic or alkaline pH_*o*_ had weaker effects on currents mediated by the R307H (I_*pH6.5*_/I_*pH7.4*_ 0.68 ± 0.04, *n* = 7; I_*pH8.0*_/I_*pH7.4*_ = 1.25 ± 0.028, *n* = 6) (Figure 6, middle) and Q1158H (I_*pH6.5*_/I_*pH7.4*_ = 0.75 ± 0.04, *n* = 7; I_*pH8.0*_/I_*pH7.4*_ = 1.06 ± 0.022, *n* = 9) (Figure 6, right) Cav3.3 channel variants.

Finally, inactivation kinetics of Cav3.3 WT and novel histidine variants were compared at the three explored pH_*o*_ (Figure 6C). The inactivation time constants measured (from the same cell) at neutral and either acidic or alkaline pH_*o*_ (τ_inact_ pH X/τ_inact_ pH 7.4) did not differ for WT- and R307H-mediated currents, whereas the inactivation kinetics of Q1158H were slowed by alkalinization (τ_inact_ pH 8.0/τ_inact_ pH 7.4 = 1.38 ± 0.05, *n* = 6, Paired test-test *p* = 0.0005, Figure 6C). Importantly, extracellular alkalinization uniquely affected the inactivation kinetics of the Q1158H variant and not WT or R307H currents (one-way ANOVA with the Tukey’s multiple comparisons test WT vs. Q1158H, *p* = 0.0017).

## Discussion

A variety of neurodevelopmental and neurological disorders are characterized by excessive neocortical cellular excitability. Migraine aura attacks are thought to be initiated by the progression of hyperexcitability to cortical spreading depression (Rogawski, 2008) and can be caused by Cav channel dysfunction, e.g., *CACNA1A* Cav2.1 mutations which cause FHM. With the genetic basis of many HM cases unknown, we undertook a WES of a large cohort of clinically diagnosed probands to investigate additional causal genes and variants. Focusing on Cav channels in this study, we found a statistically significant burden of rare missense variants in *CACNA1I* Cav3.3 in HM probands when compared to gnomAD_NFE and UK Biobank populations. The detailed electrophysiological analysis of selected Cav3.3 variants showed a range of functional effects including altered expression, changes in voltage-dependent parameters, and macroscopic inactivation kinetics, as well as modulation by pH_*o*_, suggesting they could therefore contribute to HM pathophysiology.

### R111G and D302G: Charge Neutralizations in the Extracellular Loops of Cav3.3-DI

Cells expressing Cav3.3-R111G and -D302G displayed lower current densities in comparison with Cav3.3-WT (Figure 1). Interestingly, a current density quantification of the two time points assessed revealed variant-intrinsic dynamics in the appearance of functional channels at the plasma membrane (Supplementary Figure 1). Notably, for R111G, abundant full-length protein expression could be detected by immunocytochemistry 24 h post-transfection, yet measurable currents were only present after 48 h compared to all other Cav3.3 variants studied here. R111 is conserved in all T-type Cav channels while D302 is not, yet both residues are distantly located from channel regions known to be important for gating and/or permeation that could hint at effects on open probability or single-channel conductance. As transfection and recordings of WT and these variants were performed under identical experimental conditions, their immunofluorescence patterns suggest that their delayed and/or lower current densities may be related to impairment trafficking to the plasma membrane. Both R111G and D302G also mediated slightly smaller window currents than WT Cav3.3 (Figure 4). Thus, the Cav3.3-dependent network activity in individuals carrying p.R111G and p.D302G variants may be compromised.

### M128L: A Conservative Change in the Middle of Cav3.3’s DI-S2

The expression of the Cav3.3-M128L variant was highly variable (Figure 1B) but nevertheless displayed subtle shifts in voltage-dependent activation and inactivation that translated into enlarged window currents (Figure 4). Hence, although a conservative change in DI-S2, p.M128L may lead to a subtle GOF by which a larger fraction of Cav3.3 channels support the neuronal depolarization in patients with HM.

### R307H and Q1158H: Cav3.3 Histidine Replacement Variants

Several functional properties of R307H and Q1158H Cav3.3 channels distinguished them from WT. Paradoxically, R307H and Q1158H had apparent lower current densities, yet substantially slower current kinetics and altered activation and/or inactivation voltage-dependent parameters. In their study of the schizophrenia-associated *CACNA1I* histidine replacement variant R1346H, Ghoshal et al. (2020) also noted a reduction in current density and associated changes in burst frequency (Astori et al., 2011). Nevertheless, a limitation of transient expression approaches is that the contribution of seemingly opposing effects cannot be accurately evaluated.

It has been proposed that mutations that lead to enhanced Cav3 activity such as slower inactivation can tip neuronal balance toward hyperexcitability (Perez-Reyes, 2003). An extreme example of GOF is represented by mutations in *CACNA1G* associated with early-onset cerebellar atrophy, in which A961T and M1531V mutations in Cav3.1’s from DII-S6 and DIII-S6 (respectively) result in drastically slower current inactivation kinetics and a –10 mV shift in the voltage dependence of SSI (Chemin et al., 2018). In this study, we observed that Cav3.3-R307H and -Q1158H channels inactivated slower than WT-Cav3.3. Q1158H channels display a depolarizing shift in the range of potentials at which Cav3.3 currents are available that, together with slower inactivation kinetics than WT, lead to an overall > 30% increase in its mediated window currents. In GABAergic nucleus reticularis thalamic (NRT) neurons, enhanced Cav3.3 window currents would likely contribute to an excitability imbalance.

Robust rhythm generation in the thalamus requires timely activation of T-type channels to stimulate the sodium channel activation and is imperative to processes such as synaptic plasticity in thalamic neurons (Jacquerie and Drion, 2021). The physiological role of thalamic neurons *I*_*w*_ and its contribution to the activities of other neuronal types underlines the importance of alterations in T-type channel function in neurological and psychiatric disorders (Crunelli et al., 2005). Changes in Cav3 *I*_*w*_ can contribute to pathophysiological conditions (Tsakiridou et al., 1995) directly, or as co-occurring with other abnormalities, such as extracellular pH changes (Shah et al., 2001), hyperpolarization-activated current and HVA Ca^2+^ channel variation, or sustained hyperexcitability disorders (Crunelli et al., 2005). Abnormal electrical oscillations of the cortico-thalamo-cortical network have been shown to underlay childhood absence epilepsy (CAE) and other idiopathic forms of epilepsy (Gobbo et al., 2021), while dysfunction to the thalamocortical network caused by altered Cav3.3 activation that increases tonic firing has been linked to absence epilepsy (Lee et al., 2014).

### Cav3.3-WT, R307H, and Q1158H Channels Are Differentially Modulated by Extracellular pH

Consistent with the effects of extracellular pH on ventrobasal thalamic complex relay neurons (Shah et al., 2001), extracellular alkalinization reversibly increased Cav3.3 WT currents, whereas extracellular acidification decreased it (Figure 6). Similar to Cav3.3-WT mediated currents, acidification and alkalinization of pH_*o*_ decreased and increased the Cav3.3-R307H conductance, respectively, without changes to open channel kinetics. Nevertheless, pH_*o*_ effects were somewhat attenuated in this variant despite a net increase in “protonatable” histidines in the DI p-loop (from 3 in WT to 4 in R307H). Previous studies have suggested that the protonation states of pore loops serve as extracellular redox state sensors (Li and Xu, 2012).

In stark contrast to WT Cav3.3 channels, the current amplitude of Q1158H was resistant to pH_*o*_ changes and its open-channel inactivation kinetics were slowed by extracellular alkalinization. Activity-dependent extracellular pH transients have been proposed to modulate LVA calcium currents which in turn modify the activity patterns of the thalamocortical relay neurons (Shah et al., 2001). The factors involved in the control of network excitability and synchronicity include interstitial fluid homeostasis. Thus, pH_*o*_-dependent modulation of Cav3.3 channels may support the physiological synchronization of neuronal activity. The attenuation or loss of pH_*o*_ sensitivity observed in the histidine-bearing variants R307H and Q1158H may relate to the pathophysiology of HM due to the absence of homeostatic changes in Cav3.3 conductance required in response to extracellular pH fluctuations. Extracellular pH modulation of LVA calcium currents is still incompletely understood, yet the underlying mechanisms may involve screening of outer-facing charges within the voltage sensor or pore modules of the channel protein that can shift voltage-dependent functional parameters or interfere with current flow, respectively (Delisle and Satin, 2000). To our knowledge, this study constitutes the first report of pH_*o*_ modulation of Cav3.3-mediated currents and warrants further research addressing the modulatory mechanisms of extracellular pH on the biophysical properties of Cav3.3 channels and its variants.

Cav3.3 can interact with other proteins that can modulate its channel activity including calmodulin (Chemin et al., 2017), Galpha(q/11)-coupled muscarinic acetylcholine receptors (Hildebrand et al., 2007), and the α2δ-like Cache Domain-Containing 1 (CACHD1) protein (Cottrell et al., 2018). The location of the variants functionally tested in this study did not overlap with the known binding sites on Cav3.3 for any of these proteins, although it is possible that the variants may affect the Cav3.3 function *via* unknown binding sites for these proteins or other interacting proteins.

### Functional Changes in Cav3.3 Variants Align With Predicted Deleteriousness and Potentially Interact With Additional Genetic or Environmental Factors

For *CACNA1I*, pathogenicity prediction has been shown to correlate strongly with functional effects only for rare variants (Heyne et al., 2020). The altered biophysical properties we observed for Cav3.3-Q1158H channels were consistent with *in silico* predictors (five out of six tools predicted deleterious/damaging effects). However, while significantly increased in our HM cohort, the Q1158H variant is nevertheless present in general population databases at greater frequency than the estimated prevalence of HM (∼1:10,000) (Lykke Thomsen et al., 2002), suggesting that such variants may act in combination with other susceptibility variants. For instance, in 4 of the 187 patients (2.1%) of our HM cohort, the hereto reported *CACNA1I* variants co-occurred with variants in other genes that have been implicated in HM including *PRRT2*, *PKND*, and *ATP1A4* (Sutherland et al., 2020). Thomsen and Olesen (2004) hypothesized that sporadic HM may effectively be extreme migraine with aura, as relatives of people with sporadic HM had increased risk of migraine themselves. Additionally, polygenic risk scores were found to have a stronger effect in families with migraine, and particularly HM, compared to migraineurs collected at a population level (Gormley et al., 2018). Furthermore, a recent study of GOF variants in Cav3.3 channel-gating residues demonstrated a correlation between graded effects in channel function and the severity of neurodevelopmental disorders (El Ghaleb et al., 2021). These results, along with the spectrum of functional changes observed in our assays, are consistent with the possibility that some *CACNA1I* variants may contribute to the development of hemiplegic symptoms directly, but others require additional genetic or environmental interactions/triggers (Hansen et al., 2011) to do so consistently, thus occupying a position between the monogenic causative FHM mutations and the multitude of polygenic variants involved in common forms of migraine.

The *CACNA1I* gene has been implicated in a range of disorders. *CACNA1I* GOF channel-gating mutations can cause neurodevelopmental disorders (El Ghaleb et al., 2021). *CACNA1I* is highly expressed in the dendrites of TRN neurons, where they are involved in rebound bursting (Astori et al., 2011; Lee et al., 2014). Computational modeling of relatively small Cav3.3 current reduction in TRN neurons results in complete abolishment of rebound bursting without changes in the depolarization-induced activity (Andrade et al., 2016) that is consistent with the absence of rebound bursting in knockout *CACNA1I* mice (Astori et al., 2011). Such loss of rebound bursting is predicted to reduce the thalamoreticular neuron inhibitory input over the thalamocortex, leading to increase or dysfunctional activity of the thalamus which is important for pain processing in migraine (Younis et al., 2019). Interestingly, *CACNA1I* knockout mice lacking TRN rebound bursting also evidence disrupted sleep spindles (Astori et al., 2011), while sleep disorders and sleep deprivation constitute known migraine triggers (Bigal and Lipton, 2008; Odegard et al., 2010; Wober and Wober-Bingol, 2010). As such, *CACNA1I* variants proposed to contribute to sleep rhythms (Astori et al., 2011; Ghoshal et al., 2020) are also implicated in the development of complex neuropsychiatric disorders including autism and schizophrenia (Lu et al., 2012; Andrade et al., 2016; Lory et al., 2020; El Ghaleb et al., 2021). Rare functional LOF Cav3.3 variants have been suggested to reduce the risk of schizophrenia (Baez-Nieto et al., 2021), and there is some overlap and concurrence of the functional data with the variants investigated here. Notably, migraine shows a slightly negative correlation with schizophrenia in a study exploring correlations between a wide range of psychiatric and neurological data (Brainstorm et al., 2018).

In simplified disease settings involving Cav channels, variants are classified as either GOF or LOF, depending on whether the net channel conductance is larger or smaller. However, variants often display parallel changes in several functional properties with potentially opposing effects, such as low current density/expression (LOF) and slower inactivation kinetics (GOF). Estimating which of these functional changes dominates within a physiologically relevant context will require extensive interdisciplinary approaches. Indeed, the variety of effects seen in functional assessments of mutations in our work in hemiplegic migraine, as well as studies into schizophrenia and neurodevelopmental disorders, show that even relatively similar functional changes can lead to different outcomes (Ghoshal et al., 2020; El Ghaleb et al., 2021). Given the intrinsic limitations of heterologous protein expression and window current quantification, the results reported here constitute the basis for follow-up investigation in model systems, such as variant bearing iPSC-derived corticothalamic or trigeminal neurons. Coupling functional studies, e.g., dynamic clamp, action potential clamp, and direct measurement of window currents using slow ramp protocols, in diseased and isogenic wild-type cell lines will shed further light on the physiological significance of the functional changes elicited in the *CACNA1I* variants characterized here.

In summary, we present evidence to support the hypothesis that Cav3.3 dysfunction from rare variants may contribute to the etiology of HM. The distribution of Cav3.3 and their influential role in regulating the thalamocortical network suggests this could be *via* their contributions to regulation of the neuronal excitability, sleep homeostasis, and/or perhaps in response to environmentally triggered pH disturbances. This work will potentially lead to improved molecular diagnosis of HM and understanding of its mechanisms, as well as expansion of treatment avenues to improve quality of life for patients.

## Data Availability Statement

The datasets presented in this article are not readily available because consent restrictions prevent sharing of full datasets, and the consents do not cover the deposition of WES diagnostic data into a public database. The authors declare that all data supporting the findings of this study are available within the manuscript. Requests to access the datasets should be directed to LG, lyn.griffiths@qut.edu.au.

## Ethics Statement

The studies involving human participants were reviewed and approved by Human Research Ethics Committee of the Queensland University of Technology (QUT; approval number: 1800000611). Written informed consent from the participants’ legal guardian/next of kin was not required to participate in this study in accordance with the national legislation and the institutional requirements.

## Author Contributions

LG, DA, and MC conceived and designed the project. LG, RS, and NM contributed to the patient recruitment. NM and OI performed the whole exome sequencing. NM, OI, HS, RL, and KT were involved in the analysis of genomic data. CB, RF-U, and JM performed the immunofluorescence, functional experiments, and data analyses. LH, RF-U, MC, DA, and LG supervised the research. NM, CB, HS, RS, LH, MC, RF-U, DA, and LG contributed to writing and editing. All authors contributed to the final article and approved the submitted version.

## Conflict of Interest

The authors declare that the research was conducted in the absence of any commercial or financial relationships that could be construed as a potential conflict of interest.

## Publisher’s Note

All claims expressed in this article are solely those of the authors and do not necessarily represent those of their affiliated organizations, or those of the publisher, the editors and the reviewers. Any product that may be evaluated in this article, or claim that may be made by its manufacturer, is not guaranteed or endorsed by the publisher.

## Footnotes

1
http://www.broadinstitute.org/igv

2
https://www.ncbi.nlm.nih.gov/snp/

3
www.1000genomes.org

4
https://gnomad.broadinstitute.org/

## Supplementary Material

Supplementary Figure 1 **(A)** Orthogonal display of immunostained Cav3.3 HM-associated variants. **(B)** Primary antibody (anti-flag) omitted of WT transfected and stained cells. In **(A,B)**, green corresponds to Alexa 488 fluorescence and blue is DAPI nuclear counterstain. **(C)** Current density recorded 24 h (left) and 48 h (right) post-transfection (see section “Materials and Methods”) of individual cells at each time point and their mean ± SEM. Statistical significance for data obtained at each time point was determined by one-way ANOVA with the Dunnett’s multiple comparisons test against Cav3.3 WT. *p* ≤ 0.0001 (^****^), *p* ≤ 0.005 (^**^), or *p* ≤ 0.05 (*).
