# Supplementary figures and images for: Investigation of CACNA1I Cav3.3 Dysfunction in Hemiplegic Migraine
Source: Front Mol Neurosci. 2022 Jul 19;15:892820. doi: 10.3389/fnmol.2022.892820 (PMC9345121; doi:10.3389/fnmol.2022.892820)

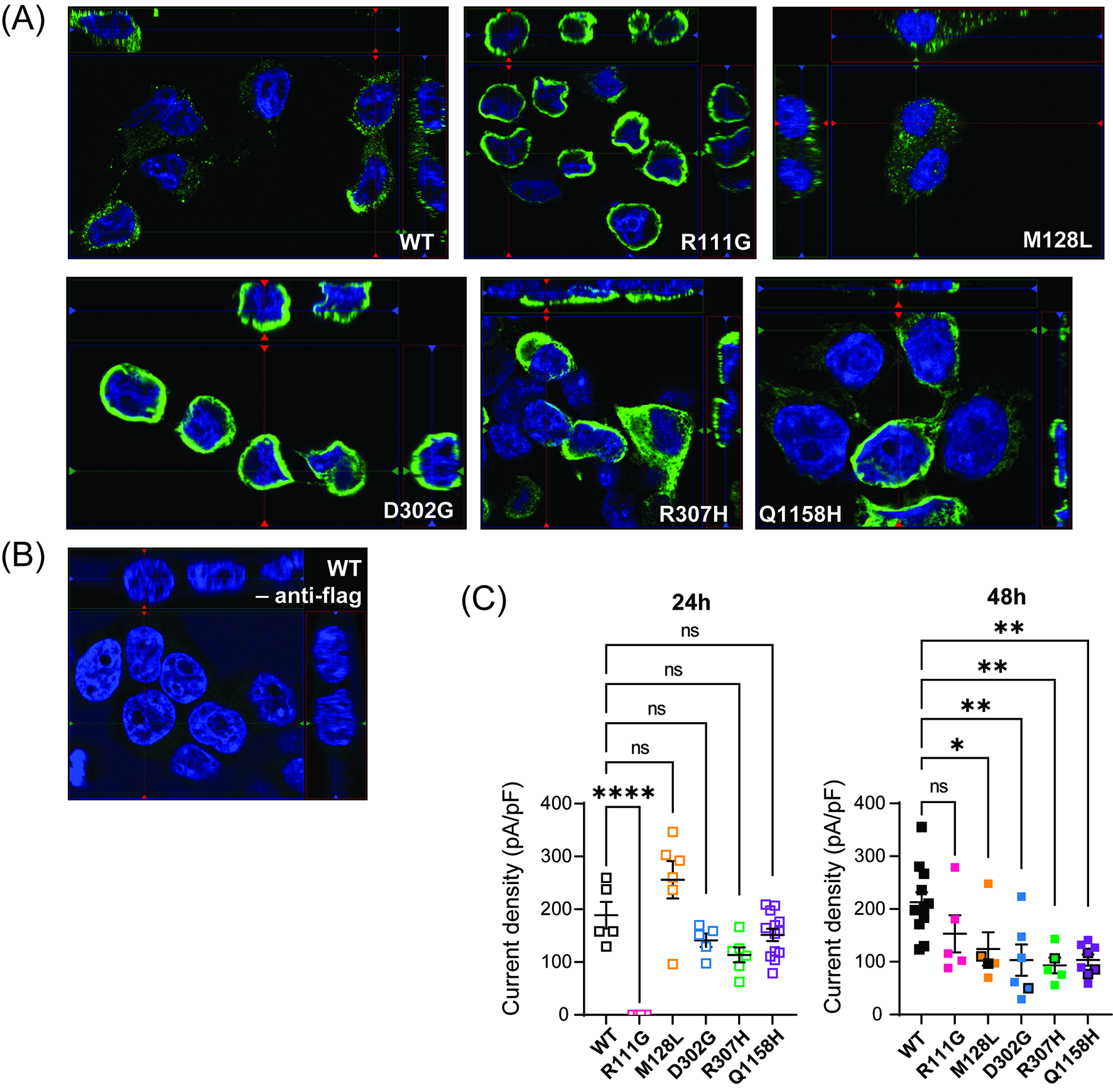

Supplement: Supplementary Figure 1 — (A) Orthogonal display of immunostained Cav3.3 HM-associated variants. (B) Primary antibody (anti-flag) omitted of WT transfected and stained cells. In (A,B), green corresponds to Alexa 488 fluorescence and blue is DAPI nuclear counterstain. (C) Current density recorded 24 h (left) and 48 h (right) post-transfection (see section “Materials and Methods”) of individual cells at each time point and their mean ± SEM. Statistical significance for data obtained at each time point was determined by one-way ANOVA with the Dunnett’s multiple comparisons test against Cav3.3 WT. p ≤ 0.0001 (****), p ≤ 0.005 (**), or p ≤ 0.05 (*). [file Image_1.TIF]
